# Effects of Thyroid Status on Regional Brain Volumes: A Diagnostic and Genetic Imaging Study in UK Biobank

**DOI:** 10.1210/clinem/dgaa903

**Published:** 2020-12-04

**Authors:** Tom Chambers, Richard Anney, Peter N Taylor, Alexander Teumer, Robin P Peeters, Marco Medici, Xavier Caseras, D Aled Rees

**Affiliations:** 1 MRC Centre for Neuropsychiatric Genetics and Genomics, Cardiff University, Cardiff, UK; 2 Cardiff University Brain Research Imaging Centre (CUBRIC), Cardiff University, Cardiff, UK; 3 Systems Immunity Research Institute, School of Medicine, Cardiff University, Cardiff, UK; 4 Institute for Community Medicine, University Medicine Greifswald, Germany; 5 Department of Internal Medicine and Academic Center for Thyroid Diseases, Erasmus Medical Center, Rotterdam, The Netherlands; 6 Department of Epidemiology, Erasmus Medical Center, Rotterdam, The Netherlands; 7 Department of Internal Medicine, Radboud University Medical Center, HB Nijmegen, The Netherlands; 8 Neuroscience and Mental Health Research Institute, School of Medicine, Cardiff University, Cardiff, UK

**Keywords:** thyroid, cerebellum, MRI, genetics, polygenic

## Abstract

**Background:**

Thyroid hormone is essential for optimal human neurodevelopment and may modify the risk of attention-deficit/hyperactivity disorder (ADHD). However, the brain structures involved are unknown and it is unclear if the adult brain is also susceptible to changes in thyroid status.

**Methods:**

We used International Classification of Disease-10 codes, polygenic thyroid scores at different thresholds of association with thyroid traits (*P*_T_-values), and image-derived phenotypes in UK Biobank (n = 18 825) to investigate the effects of a recorded diagnosis of thyroid disease and genetic risk for thyroid status on cerebellar and subcortical gray matter volume. Regional genetic pleiotropy between thyroid status and ADHD was explored using the GWAS-pairwise method.

**Results:**

A recorded diagnosis of hypothyroidism (n = 419) was associated with significant reductions in total cerebellar and pallidum gray matter volumes (β [95% CI] = −0.14[−0.23, −0.06], *P* = 0.0005 and β [95%CI] = −0.12 [−0.20, −0.04], *P* = 0.0042, respectively), mediated in part by increases in body mass index. While we found no evidence for total cerebellar volume alterations with increased polygenic scores for any thyroid trait, opposing influences of increased polygenic scores for hypo- and hyperthyroidism were found in the pallidum (*P*_T_ < 1e−3: β [95% CI] = −0.02 [−0.03, −0.01], *P* = 0.0003 and *P*_T_ < 1e−7: β [95% CI] = 0.02 [0.01, 0.03], *P* = 0.0003, respectively). Neither hypo- nor hyperthyroidism showed evidence of regional genetic pleiotropy with ADHD.

**Conclusions:**

Thyroid status affects gray matter volume in adults, particularly at the level of the cerebellum and pallidum, with potential implications for the regulation of motor, cognitive, and affective function.

Thyroid hormone (TH) is essential for optimal neurodevelopment, exemplified by the severe intellectual disability that affected patients with untreated congenital hypothyroidism prior to the introduction of neonatal screening. TH influences several aspects of neurodevelopment, including differentiation and proliferation of neuronal precursors, neuronal migration, and myelination (1). Early pregnancy may be a critical window, since suboptimal maternal gestational thyroid function has been implicated as a risk factor for neurodevelopmental disorders (2, 3), while behavioral difficulties may affect children born to mothers exposed to excess thyroxine (T4) in pregnancy (4).

The brain regions involved in any potential effects of TH, however, are unclear, although the cerebellum is emerging as a major target. Cerebellar ontogenesis is profoundly influenced by TH: in rodents, hypo- and hyperthyroidism affect foliation, differentiation, and migration of granule cells; Purkinje cell arborization; and neuronal cell death (1). Such effects might provide a mechanistic link to the risk of neurodevelopmental disorders, since the cerebellum and subcortical connections are implicated as core structures in the pathogenesis of ADHD (5).

However, human structural brain studies in thyroid disease are limited. In a prospective study of >1900 mother-child pairs from the Generation R cohort, an inverse U-shaped association was noted between maternal free T4 levels and offspring gray matter volume (6), suggesting that both low and high TH levels may adversely affect human neurodevelopment. Altered thyroid status may also affect gray matter volume in adults (7, 8), although the numbers studied to date are small.

A new resource for relating recorded diagnoses and genetics to neuroimaging is UK Biobank. This prospective epidemiological study of 500 000 middle-aged volunteers (9) includes structural brain magnetic resonance imaging (MRI) of regional gray matter volumes. We thus set out to investigate whether a recorded diagnosis of hypo- or hyperthyroidism in UK Biobank was associated with alterations in gray matter volume in adults, hypothesizing that any effects observed would be opposing, and most apparent in the cerebellum. We additionally sought to explore whether genetic risk for thyroid disorders had similar effects on gray matter volume, and whether such a risk overlapped with that for ADHD.

## Methods

### Participants

The UK Biobank is a population-based, volunteer cohort of 500 000 participants (40-69 years of age at recruitment) who have provided extensive medical, lifestyle, and genetic data (9). Our study used the brain MRI imaging-derived phenotype (IDP) data of 22 000 participants who had been scanned by UK Biobank (2014-2018) and had their data released by the time of study initiation. Using International Statistical Classification of Diseases and Related Health Problems revision-10 (ICD-10) primary and secondary diagnostic codes from participants’ hospital inpatient records (from 1996 to present) and from self-reports of medical conditions (provided at UK Biobank centre), we removed individuals with severe neurological and psychiatric conditions (Supplementary note 1 (10)). Using the thyroid-relevant hospital inpatient ICD-10 code recordings (E00-E07), we derived a categorical variable of having had anytime recorded diagnoses for hypothyroidism, hyperthyroidism, or other thyroid disorders (Supplementary note 1 (10)). For genetic analyses, we further removed those subjects with ICD-10 or self-reported thyroid disorder. Additional imaging and genetic exclusion criteria and quality control are outlined below. Ethical approval for UK Biobank was granted by the North West Multi-Centre Ethics Committee. Data for this study were obtained under application number #17044.

### MRI data

A full description of the imaging acquisition, quality control and imaging-derived phenotype (IDP) generation from UK Biobank can be found elsewhere (11). Briefly, UK Biobank participants attended 1 of 2 imaging sites using the same scanner design (3-Tesla Siemens Skyra scanner; 32-channel head coil) and underwent an extensive imaging protocol, which included a T1-weighted structural scan (3D Magnetization Prepared Rapid Acquisition Gradient Echo with 1mm^3^ isotropic resolution), whose data we used here. We used the released IDP produced by UK Biobank using processing tools from FMRIB (Functional Magnetic Resonance Imaging of the Brain) Software Library (12) (FSL), including FAST (13) (FMRIB’s Automated Segmentation Tool) registration of cerebellar lobules atlas and FIRST (14) (FMRIB’s Integrated Registration and Segmentation Tool) registration of subcortical regions atlas. Of the cerebellar lobule IDPs, we excluded Crus I vermis due to its small size and likely low signal-to-noise ratio. We grouped these regions of interest by hemisphere, and all cerebellar lobules into a single total cerebellar volume measure, analyzing any lobule-specific effects as secondary analyses. We chose this approach because no cerebellar-specific registration process was applied, likely reducing the signal-to-noise ratio in the cerebellum, because cerebellar lobules are highly correlated and because the lobule boundaries show only small association with functional boundaries, meaning that detection of strong lobule-specific effects was deemed less likely. Subjects with any covariate imaging value (see below) deemed as outliers (>5 × median absolute deviation from the overall median) were removed from further analysis and, of these, those with remaining outlier values were excluded from each respective region analysis (Supplementary Table 1 (10)).

### Genetic data

A full description of UK Biobank’s data collection, quality control, and imputation process can be found elsewhere (http://www.ukbiobank.ac.uk/scientists-3/genetic-data/). Locally, we further applied additional quality control of the imaging-sample raw genotypes using the *genotypeqc* function (https://github.com/ricanney/stata). All Stata functions described leverage PLINK (15) (v1.90b5.4; www.cog-genomics.org/plink/1.9/). Briefly, all markers were harmonized to genome build hg19 and common nomenclature was applied based on the Haplotype Reference Consortium (HRC) r1.1. We excluded markers based on individual missingness (>2%), low minor allele count (<5), deviations from Hardy-Weinberg equilibrium (*P* < 10^–10^), and from expected minor allele frequency (MAF; defined as 4 SD from the reported 1000 Genomes Project phase-3 GBR MAF). Participants were excluded based on their overall marker missingness (>2%) and marker heterozygosity (>4 × SD from sample mean). Using the *bim2ancestry_keep* function (https://github.com/ricanney/stata), we limited to GBR ancestry (>4 × SD from 1000 Genomes Project phase-3 GBR sample mean for the first 3 principal components) and using *bim2unrelated* we removed one of each pair of close relatives (>0.0442 kinship coefficient ie, third-degree relatives). A total of 7 726 488 markers were included in downstream analysis.

Polygenic score training data were obtained from the most recent genome-wide association study (GWAS) meta-analysis on circulating thyroid hormone levels, which tested up to 72 168 participants of European ancestry and provided summary statistics for thyroid-stimulating hormone (TSH) and free thyroxine (fT4) levels in subjects with TSH levels within the cohort reference range, as well as for elevated (hypothyroidism) and reduced (hyperthyroidism) TSH compared with these ranges (16). Each thyroid disorder GWAS was based on 7 858 695 and 7 980 324 genetic markers, respectively. We utilized the summary statistics across genders, rather than sex-specific summary statistics. Summary statistics were harmonized to hg19 build and HRC reference nomenclature using *summaryqc* (https://github.com/ricanney/stata).

Polygenic scores were created using the PLINK *summaryqc2score* wrapper function (https://github.com/ricanney/stata). Regions of known high linkage disequilibrium (17) were excluded prior to calculating polygenic scores. Scores were subsequently calculated from linkage disequilibrium–independent (r^2^ < 0.2) markers for hyper- and hypothyroidism, and TSH and free-T4 level summary statistics for single nucleotide polymorphism (SNP) inclusion *P* value thresholds (*P*_T_) of *P*_T_ < 0.5, <0.1, <0.05, <0.01, <1e−3, <1e−4, <1e−5, <1e−6, <1e−7, and <1e−8. An individual’s polygenic score is a linear weighted sum of their genotype values weighted by their effect sizes for each thyroid trait.

### Statistical methods

For our analyses we used univariate multiple linear regression in R(3.6.0) (https://www.R-project.org/). Our first primary analyses investigated the effect of hypothyroid and hyperthyroid diagnoses on total cerebellar and subcortical volumes. The control group in this analysis were those with no thyroid-related diagnoses. For each model, we controlled for potential confounding by other imaging variables of head size (volumetric scaling applied for registration, analogous to the inverse of head size; 25000); imaging center attended (54-2.0); X-, Y-, and residual Z-head position in the scanner (25756, 25757, 25758, with Z-position having starting table-Z-position regressed out; 25759); along with demographic covariates of age (21003-2.0, quadratic); sex (18); and their interaction. Histograms were used to confirm normally distributed residuals. We tested for any mediation of body mass index (BMI) on overt diagnoses differences using the *mediation* package (19) with 1000 bootstrapping simulations, while also controlling for the above covariates. Using the *medsens* function (19), we tested for violations of the sequential ignorability assumption that there are no unmeasured confounders of the mediator-outcome pathway. In the subset with no overt or self-reported thyroid disorders, we repeated the above analysis using polygenic scores for thyroid-related traits. For these genetic predictors, in addition to the above covariates, we also controlled for BMI (log transformed; 21001–2.0) as well as the first 10 genetic principal components to correct for residual population structure. Secondary analyses investigated any lobule-specific effects. Standardized β coefficients reflecting changes in units of SD, corresponding 95% CI, and the variance explained in the outcome uniquely attributable to the phenotype of interest (ΔR^2^; calculated by subtracting each model R^2^ with the phenotype against those without) are provided. The Benjamini-Hochberg method (20) was used to control the false discovery rate (FDR < 0.05) for the number of models assessed in each section (thyroid diagnoses and polygenic scores).

We also aimed to investigate evidence for regional pleiotropy between ADHD and hypo- and hyperthyroidism using the GWAS-pairwise method (21) utilizing recent ADHD GWAS meta-analysis summary statistics of 20 183 ADHD cases and 35 191 controls (22). Following application of the same quality control protocols outlined above, 5 907 045 genetic markers were carried forward for analyses. We performed 2-sample meta-analysis using METAL (23), with the second pair in the GWAS performed with the reported β and with the sign of the β flipped so as to not avoid omitting those markers with opposing effects. Three models were examined; model-1 where the association is driven by thyroid trait GWAS (gwas-1), model-2 where it is driven by ADHD GWAS (gwas-2), and model-3 where it is driven by signal at both gwas-1 and gwas-2. For any marker, model-3 was accepted if *P*_model-3_ < 5e-8 and independently *P*_gwas-1_ and *P*_gwas-2_ were both observed at *P* < 1e-5.

## Results

### Effects of thyroid disorders on cerebellar and subcortical brain volumes

After exclusion, 18 825 individuals (age [mean ± SD] = 62.7 ± 7.45; male = 48%) remained (Table 1), of whom 538 (3.1%) had an ICD-10 thyroid-related disorder (n = 419 [2.2%] hypothyroidism, n = 33 [0.2%] hyperthyroidism, and n = 86 [0.5%] other thyroid disorder or a history of both hypo- and hyperthyroidism) (Table 1). Hypothyroidism cases were, on average, 2 years younger than controls (*P* = 5.09e−7), while hyperthyroidism cases showed no significant difference (*P* = 0.17) and both disorders were more common in females. Correcting for demographic and imaging covariates, head size (inverse of head size; see Methods) was not significantly altered in either hypo- or hyperthyroidism cases compared with controls (β [95% CI] = 0.023 [−0.052, 0.098], *P* = 0.55 and β [95% CI] = −0.023 [−0.493, 0.034], *P* = 0.09, respectively).

**Table 1.** Demographic Information for the Study Cohort, Including Those With ICD-10 Diagnosis of Hypothyroidism, Hyperthyroidism, Other Thyroid-Related Disorders and Those Without any Thyroid-Related Diagnosis (normative controls)

	*Hypothyroidism (n* = *419)*	*Hyperthyroidism (n* = *33)*	*Other (n* = *86)*	*No (n* = *18287)*	*Total*
***Sex***					
*F*	338 (80.7%)	29 (87.9%)	75 (87.2%)	9440 (51.6%)	9882 (52.5%)
*M*	81 (19.3%)	4 (12.1%)	11 (12.8%)	8847 (48.4%)	8943 (47.5%)
***Age (years)***					
*Mean (SD)*	64.5 (7.32)	60.8 (9.05)	63.1 (6.53)	62.6 (7.45)	62.7 (7.45)
*Median [Min, Max]*	65.0 [47.0, 78.0]	62.0 [46.0, 78.0]	64.0 [46.0, 74.0]	63.0 [45.0, 80.0]	63.0 [45.0, 80.0]
***Body mass index***					
*Mean (SD)*	28.0 (4.92)	28.6 (5.55)	26.3 (4.23)	26.6 (4.39)	26.6 (4.41)
*Median [Min, Max]*	27.3 [17.9, 49.2]	27.8 [17.9, 46.5]	25.7 [17.1, 40.2]	25.9 [13.4, 58.0]	26.0 [13.4, 58.0]
*Missing*	11 (2.6%)	2 (6.1%)	5 (5.8%)	428 (2.3%)	446 (2.4%)

**Figure 1.**
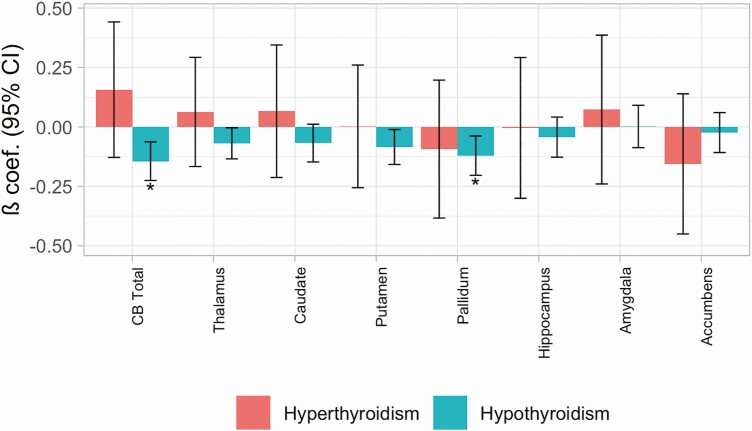
Effect on total cerebellar (CB) and subcortical volumes of a diagnosis of hypothyroidism and hyperthyroidism compared to normative controls. All values are following correction for demographic and imaging covariates. Standardized beta coefficients (β) and 95% CI are provided. *signifies results with FDR < 0.05.

For our main analyses, we tested for differences in total cerebellar and subcortical volumes in cases of hypo- and hyperthyroidism compared to controls (Table 2; Fig. 1). For hypothyroidism, we found significant (FDR < 0.05) reductions in bilateral total cerebellar and pallidum volumes (β [95% CI] = −0.14 [−0.23, −0.06], *P* = 0.001 and β [95% CI] = −0.12 [−0.20, −0.04], *P* = 0.004, respectively). There were no volume changes in hyperthyroidism cases (*P* > 0.05). Exploring the effects across the cerebellum (Supplementary Table 2 (10); Supplementary Fig. 1 (10)), we found significant reductions across most cerebellar lobules in hypothyroidism cases, aside from superior posterior vermal regions (VI-VIIIa vermis). Only one region, the Crus II hemispheric volume, indicated possible opposing effects between hypo- and hyperthyroidism (β [95% CI] = −0.11 [−0.20, −0.03], *P* = 0.02 and β [95% CI] = 0.40 [0.09, 0.71], *P* = 0.01, respectively).

**Table 2.** Effect on Total Cerebellar (CB) and Subcortical Volumes of Diagnosis of Hypothyroidism and Hyperthyroidism Compared With Normative Controls

		*ICD-10 Hypothyroidism*					*ICD-10 Hyperthyroidism*				
	*ΔR* ^*2*^	*β*	*CI.l*	*CI.u*	*P*	*FDR*	*β*	*CI.l*	*CI.u*	*P*	*FDR*
***CB Total***	4.95E-04	-0.144	-0.225	-0.063	0.001	**0.008**	0.157	-0.128	0.442	0.281	0.636
***Thalamus***	1.10E-04	-0.069	-0.134	-0.003	0.039	0.156	0.063	-0.167	0.293	0.591	0.795
***Caudate***	1.08E-04	-0.068	-0.147	0.011	0.094	0.300	0.066	-0.213	0.345	0.642	0.795
***Putamen***	1.55E-04	-0.085	-0.158	-0.011	0.024	0.129	0.002	-0.256	0.260	0.986	0.986
***Pallidum***	3.30E-04	-0.121	-0.204	-0.038	0.004	**0.034**	-0.093	-0.383	0.197	0.529	0.795
***Accumbens***	5.42E-05	-0.024	-0.108	0.060	0.579	0.795	-0.155	-0.450	0.140	0.302	0.636
***Hippocampus***	3.99E-05	-0.043	-0.127	0.041	0.318	0.636	-0.004	-0.300	0.292	0.979	0.986
***Amygdala***	9.49E-06	0.002	-0.087	0.091	0.966	0.986	0.073	-0.240	0.386	0.646	0.795

All values are following correction for demographic and imaging covariates in the model. Variance of volumes uniquely explained by thyroid ICD-10 hospital record variable (ΔR^2^), Standardized B-coefficients (β), 95% CIs (lower and upper CI), uncorrected *P* values and controlled false discovery rate (FDR) q-values for the number of genetic predictors are provided. Bold signifies results FDR < 0.05.

Since BMI was higher in subjects with hypothyroidism (Table 1) we tested whether it mediated any of the significant relationships observed between hypothyroidism and brain morphometry. We confirmed this for total cerebellar (40% [25%, 82%] mediated, *P* < 2e−16) and pallidum volume (23% [12%, 61%] mediated, *P* < 2e−16) although significant average direct effects (ADE) remained (Supplementary Table 3 (10)). The results, however, were sensitive to violations of sequential ignorability, and if relatively small residual correlation between BMI and brain volumes exist, then these would make this assumption invalid (rho > −0.2 and −0.1 for cerebellar and pallidal analyses).

### Effects of genetic risk for thyroid disorders on cerebellar and subcortical brain volumes

**Figure 2.**
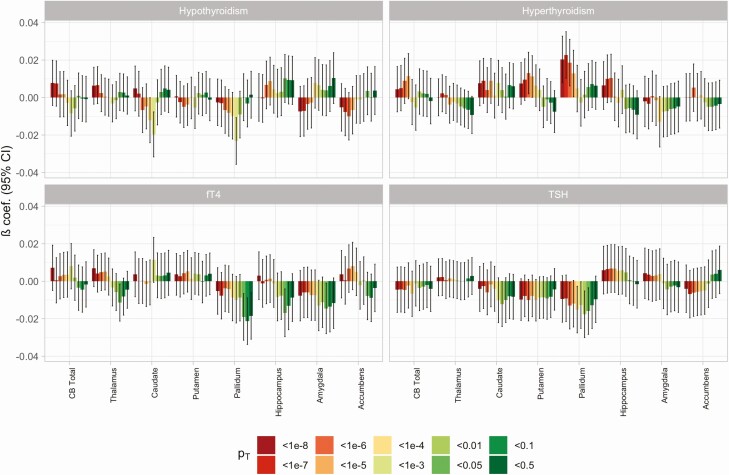
Effect on total cerebellar (CB) and subcortical volumes of increasing polygenic score (PGS) for hypothyroidism, hyperthyroidism, free thyroxine (fT4) and thyroid-stimulating hormone (TSH) across the different SNP *P* value inclusion thresholds (*P*_T_). All values are following correction for demographic, genetic, and imaging covariates. Standardized beta coefficients (β) and 95% CI are provided. Of note, no results were below our FDR < 0.05 threshold.

For our genetic analyses, we removed ICD-10 or self-reported cases of any thyroid-related diagnoses, leaving N = 18 255 subjects (Supplementary Table 4 (10)). We tested for the effect of increasing thyroid polygenic score for hypo- and hyperthyroidism, and TSH and fT4 levels on cerebellar and subcortical volumes, controlling the FDR for the number of volumes, thyroid traits, and SNP *P* value thresholds (*P*_T_-values) assessed. The number of GWAS-significant SNPs at each *P*_T_-value is provided in Supplementary Table 5 (10). We found no relationships across any thyroid trait at any *P*_T_-value reaching our significance threshold when controlling FDR < 0.05 (Fig. 2, Supplementary Table 6 (10)), although opposing effects in the pallidum of increasing polygenic scores for hyper- and hypothyroidism were close to this threshold (*P*_T_ < 1e−7: β [95% CI] = 0.02 [0.01, 0.03], *P* = 0.0003 and *P*_T_ < 1e−3: β [95% CI] = −0.02 [−0.03, −0.01], *P* = 0.0003, respectively). This included finding no evidence for total cerebellar volume alterations with increased polygenic score for any thyroid trait (*P* > 0.05). While this was also reflected at the cerebellar lobule level for most thyroid traits, there was a fairly consistent pattern of lobule volume increases at more stringent hyperthyroidism polygenic score *P*_T_-values, although none reached our significance threshold following multiple comparison correction (Supplementary Table 7 (10); Supplementary Fig. 2 (10)).

### Genetic association between thyroid disorders and ADHD

Finally, we investigated evidence for regional genetic pleiotropy between common genetic variants important for our thyroid disorders and those for ADHD disorder. Using GWAS-pairwise analyses, we found that neither hypothyroidism nor hyperthyroidism showed any evidence for regional genetic pleiotropy with ADHD; with no SNPs observing model-3 *P*_model-3_ < 5e−8, while also observing both *P*_gwas-1_ and *P*_gwas-2_ < 1e−5.

## Discussion

This is the first study to have examined an effect of thyroid status on regional brain volumes in adults on a large scale. We used a combination of a recorded diagnosis and genetic risk scores to confirm an effect of thyroid status on gray matter volume, notably at the level of the cerebellum and pallidum.

A recorded diagnosis of hypothyroidism was associated with modest reductions in cerebellar and pallidum volume when controlling for overall head size. These findings are consistent with clinical observations of cerebellar dysfunction in hypothyroidism and Hashimoto’s encephalopathy (24), as well as rodent studies demonstrating high TH receptor expression in the cerebellum during development (25) and TH effects on cerebellar cell differentiation, arborization, migration, and death (1). In addition to its well-established roles in motor coordination, the cerebellum also regulates various cognitive and affective processes (26). These are mediated via widespread connections to cortical and subcortical regions, including direct connections with the pallidum (27) and other structures within the basal ganglia. The volume reductions in hypothyroidism were evident across cerebellar lobules, with the Crus II region, a nonmotor cerebellar region with high basal ganglia connectivity (27), being the only lobule showing both hypothyroidism-related reductions and hyperthyroidism-related increases in volume. The findings of our study thus indicate that disruption to cerebellar-pallidal pathways might be a key neuronal feature of thyroid disorders.

We found no change in hypothyroidism cases in other subcortical regions or in head size derived from the scaling applied to T1 images. These findings differ from those of a recent study, in which 70 adults with elevated TSH had significantly lower total brain volume than euthyroid controls, while low TSH was not associated with change in brain volume (28). In both cases, total gray matter volume was unaffected by TSH status. A reduction in hippocampal volume in subjects with elevated TSH was also apparent in this and another study (29). Since we did not observe any alterations in hippocampal or total volume, these differences may suggest that hippocampal effects occur only in active disease and resolve with appropriate treatment. Alternatively, the differences may relate to our larger sample size, different populations, or the diagnoses used. We did not find any significant alterations in subjects with a diagnosis of hyperthyroidism, although the volume effects were broadly opposite to hypothyroidism and we were likely underpowered in view of the much smaller subject numbers.

Since hypothyroidism is associated with weight gain, we undertook mediation analysis to examine the influence of BMI, finding that the effects of hypothyroidism on gray matter volume reduction were mediated in part by BMI. To our knowledge, this finding has not been reported previously, although increased BMI is recognized as a risk factor for gray matter volume loss in in older age (30). Our findings may thus have important implications for physicians managing patients with hypothyroidism, placing an emphasis on minimization of weight gain to protect against gray matter volume loss. However, since our analysis was retrospective, further studies assessing causality should be performed. For instance, in addition to BMI, an effect of elevated weight on reducing MRI signal-to-noise ratio, including via increased head motion (18, 31), cannot entirely be excluded and should be explored in future studies, in addition to the effect of smoking status, which is known to influence thyroid function (32) as well as affecting brain volume (33). Since direct effects on volume loss still remained when controlling for BMI, other mechanisms may also be in operation, including reduced T3 signaling, as patients established on levothyroxine replacement for hypothyroidism display reduced serum T3 levels despite normalization of TSH (34).

We undertook our genetic substudy with an aim of replicating findings from recorded diagnosis. We found little evidence for an effect of polygenic scores on gray matter volume in most regions of interest, with the exception of the pallidum in which opposing influences of polygenic scores for hypo- and hyperthyroidism were found, albeit of borderline significance. However, it should be recognized that only a relatively small proportion of the variance in thyroid function is explained by the latest GWAS, with reduced power especially for increased or decreased TSH, since subjects with known thyroid disease were excluded. Nevertheless, the effects on gray matter volume in the pallidum raise the possibility of a shared influence of genetic risk for hypo- and hyperthyroidism with gray matter volume in this subcortical region. Large-scale genetic imaging analysis has led to the discovery of several genetic variants important for subcortical development (35) and our results indicate that exploration of genomic loci showing pleiotropy for both pallidal development and thyroid disorder might prove fruitful.

Finally, in light of several studies suggesting a link between thyroid status and ADHD risk, and common morphometric alterations at the level of the cerebellum (5), we sought to establish if there was any genetic evidence for pleiotropy between thyroid disorders and ADHD using GWAS-pairwise analyses. Since we found no evidence for pleiotropy, this may imply that any excess risk of ADHD related to disturbed thyroid status relates to environmental influences, such as altered maternal thyroid function, rather than shared genetic etiology. Our recent observations of an increased risk of behavioral disturbances in children born to mothers exposed to excess thyroxine replacement in pregnancy (4) supports the view that the *in utero* environment is a critical window. This risk may be mediated, at least in part, through excess TH receptor alpha signaling in view of the high prevalence of ADHD reported in children with resistance to thyroid hormone beta (36).

Our study has a number of strengths, including the large sample size and integrated genetic and imaging analysis. This provides a template by which future studies could be undertaken, for example when the imaging dataset in UK Biobank extends to its intended target of over 100 000 participants. However, our study also has several limitations, including a lack of thyroid function tests, which necessitated our use of recorded diagnosis and polygenic scores as markers of thyroid status. The prevalence of hypothyroidism was also lower than the most recent UK data. We suspect that this may relate to differences in sociodemographic and health-related characteristics of participants in UK Biobank, who are more likely to be older, live in less socioeconomically deprived areas, and have fewer self-reported health conditions (37). We also confined our analyses to gray matter volume; since TH also affects myelination, future studies might also include an assessment of white matter volume, especially in childhood and adolescence when the expansion in myelin deposition is profound. In addition, as with almost all genetic imaging analyses, we found that the variance explained in our volumes by genetic risk scores was relatively low. Methods to improve this in future studies, including larger samples, more complex genetic analyses (eg, gene × gene and gene × environment interactions) and use of cerebellar-specific registration tools, would be invaluable. Finally, anatomical lobules are unlikely to offer the best separation of cerebellar function, with atlases defined by resting and task-based functional MRI scans available (26, 38).

To conclude, our study provides evidence for an effect of thyroid status on gray matter volume in adults, most notably at the level of the cerebellum and pallidum. These observations extend our understanding of the influence of TH on neuronal structure in the human brain. However, further studies are needed to replicate and extend our findings, including a focus on imaging datasets in childhood and adolescence, where any effects of altered thyroid exposure on neurodevelopment might be expected to be more profound.

## Data Availability

Some or all data generated or analyzed during this study are included in this published article or in the data repositories listed in References.

## Abbreviations

ADHD: attention-deficit/hyperactivity disorder
BMI: body mass index
FDR: false discovery rate
fT4: free thyroxine
GWAS: genome-wide association study
ICD-10: International Statistical Classification of Diseases and Related Health Problems revision-10
IDP: imaging-derived phenotype
MRI: magnetic resonance imaging
T4: thyroxine
TH: thyroid hormone
TSH: thyrotropin (thyroid-stimulating hormone)

## Additional Information

***Disclosures:*** The authors declare no conflict of interest.
